# Heterogeneous ketonic decarboxylation of dodecanoic acid: studying reaction parameters†

**DOI:** 10.1039/d1ra06871g

**Published:** 2021-11-03

**Authors:** Diego D. Perera-Solis, Vladimir L. Zholobenko, Andrew Whiting, Hugh Christopher Greenwell

**Affiliations:** Department of Chemistry, Durham University Durham DH1 3LE UK diego.d.perera-solis@durham.ac.uk; Centre for Sustainable Chemical Processes, Department of Chemistry, Durham University Durham DH13LE UK; Lennard-Jones Laboratories, Keele University Staffordshire ST5 5BG UK v.l.zholobenko@keele.ac.uk; Department of Earth Sciences, Durham University Durham DH1 3LE UK

## Abstract

Ketonic decarboxylation has gained significant attention in recent years as a pathway to reduce the oxygen content within biomass-derived oils, and to produce sustainable ketones. The reaction is base catalysed, with MgO an economic, accessible and highly basic heterogeneous catalyst. Here we use MgO to catalyse the ketonic decarboxylation of dodecanoic acid to form 12-tricosanone at moderate temperatures (250 °C, 280 °C and 300 °C) with low catalyst loads of 1% (w/w), 3% (w/w) and 5% (w/w) with respect to the dodecanoic acid, with a reaction time of 1 hour under batch conditions. Three different particle sizes for the MgO were tested (50 nm, 100 nm and 44 μm). Ketone yield was found to increase with increasing reaction temperature, reaching approximately 75% yield for all the samples tested. Temperature was found to be the main control on reaction yield, rather than surface area or particle size.

## Abstract 2

Ketonic decarboxylation has gained significant attention in recent years as a pathway to reduce the oxygen content within biomass-derived oils, and to produce sustainable ketones.

## Graphical abstract

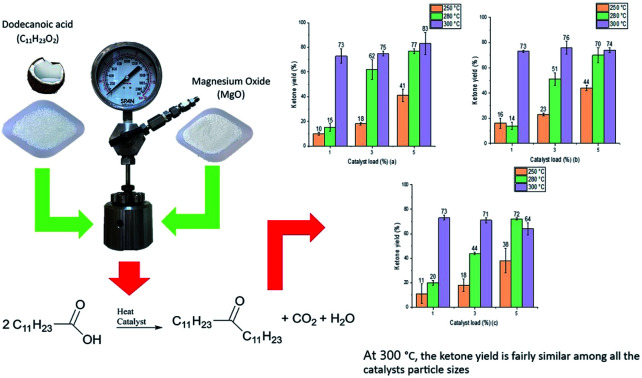

## 1. Introduction

Among renewable energy sources and sustainable provision of chemical feedstocks, biomass plays an important role. To date, biomass contributes to the production of 2 million barrels a day of transportation fuels and to 14% of the world's primary energy demand.^1,2^ Biomass is key to delivering sustainable infrastructure for the future that helps to reduce dependence on fossil fuel derivatives as well as meeting demands from society in terms of green energy. Although generally referring to organic matter coming from plants, biomass can be subdivided into wastes (*e.g.* agricultural production wastes, crop residues, urban organic wastes), forest products (*e.g.*, wood, trees, shrubs, wood residues) and energy crops (*e.g.*, starch crops, sugar crops, oilseed crops). The most important drawback of using biomass as an energy source compared to fossil fuels is its low heating value, making it less suitable for direct application for primary energy production. However, due to its high ignition stability and sustainability, biomass can be easily processed using thermochemical approaches and converted into higher value fuels.^2^ Different strategies are also being studied to transform biomass-derived oils and sugars into commodity chemicals.^1^

One class of thermochemical transformation routes for biomass-derived oils are deoxygenation reactions, in which oxygenated compounds (*e.g.* biodiesel, pyrolysis oils and fatty acids) be converted into higher energy density, lower oxygen content fuels. These reactions include decarboxylation, decarbonylation and hydro-deoxygenation, with the latter the most expensive in terms of energy requirements and the only one that involves hydrogen.^3–5^ Ketonic decarboxylation (*i.e.* ketonisation) is a deoxygenation reaction widely studied over the last decades. Increasing carbon chain length while removing oxygen in the form of carbon dioxide and water, the reaction proceeds as follows:R_1_COOH + R_2_COOH → R_1_COR_2_ + CO_2_ + H_2_O

The reaction mechanism for ketonic decarboxylation has been studied extensively in recent years, as well as the behaviour of different catalysts and substrates under different conditions.^6–14^ In our recent study,^15^ ketonisation of stearic acid in the liquid phase was explored using solid mixed metal hydroxides/oxides catalyst with promising results. The catalysts used were layered double hydroxides (LDHs) and the respective mixed metal oxides (MMOs) obtained from LDHs after calcination. Yields of the ketonisation product from stearic acid (*i.e.*, stearone) of up to 90% were obtained using the LDH at relatively moderate reaction temperatures (250 °C). The corresponding MMOs also exhibited good ketone yield of up to 80%. In the initial study the feed to catalyst ratio was high (20% w/w) and the effects of varying key parameters were not explored.

In this present study, a different catalyst has been used for the ketonisation reactions. In our earlier study, we used MgO as a control against the LDH and it was shown to also possess activity for the ketonisation reaction.^15^ Due to its strong basic properties and simplicity, magnesium oxide (MgO) is used here as a heterogeneous catalyst. Following the findings from our last study, moderate to high reaction temperatures are explored, as well as the impact of varying the catalyst to feed ratio. In addition, the effect of particle size of the catalyst on the production of the ketones was explored using three different sizes, two in the range of the nano-size (100 nm and 50 nm) and a third one within the μm range. Through evaluating the effect of particle size, and hence surface area, whether the reaction was controlled by surface reactive site availability could be probed. The effect of particle size on intrinsic reactivity of the MgO is also studied through analysis of basic site strength and distribution.

## 2. Materials and methods

All chemicals and reagents were used as received from commercial sources, without any further purification: magnesium oxide 100 nm (Alfa Aesar, 99%), magnesium oxide −325 mesh (44 μm, Alfa Aesar, 98%), magnesium oxide 50 nm (Sigma-Aldrich, 99%), dodecanoic acid (TCI, 98%) and toluene (Fischer, analytical-grade 99%).

### 2.1 Catalyst characterization

#### 2.1.1 Powder X-ray diffraction

Dried LDH samples were analysed using powder X-ray diffraction (PXRD). A Bruker D8 Advanced XRD instrument was used with a copper tube with radiation of 1.5418 Å wavelength. The 2*θ* angle range was set to be 10–90 degrees. The sample was set to run for 45 minutes total scan time with a step size of 0.02°. The MgO powder was placed on a PXRD slide through a 120 mesh (125 μm) sieve to uniformly disperse the sample over the whole surface of the slide.

#### 2.1.2 Thermogravimetric analysis (TGA)

TGA was performed using a PerkinElmer Thermogravimetric Analyzer 8000, with pyrolysis performed under N_2_ to study the thermal decomposition of the samples. The temperature was increased from room temperature to 1000 °C, at a rate of 30 °C per minute.

#### 2.1.3 Fourier transform infrared (FTIR) spectroscopy

FTIR spectroscopy was performed on a PerkinElmer FT-IR spectrometer, fitted with an attenuated total reflectance (ATR) cell, in the range 4000 to 400 cm^−1^. The force gauge was set at a consistent 105 units on the ATR cell anvil. 5 to 10 mg of sample was carefully placed on the force gauge. Fifteen scans per sample were taken.

#### 2.1.4 Surface area analysis

Pore volume, surface area and average pore size were measured using N_2_ gas on a Micromeritics ASAP 2020 system at −196 °C, with samples degassed at 80 °C.

#### 2.1.5 Scanning electron microscopy (SEM)

SEM experiments were performed using a FEI Helios Nanolab 600 with a field emission gun (FEG) source, operating at 5 kV.

#### 2.1.6 Temperature programmed-desorption (TPD) with CO_2_

TPD adsorption experiments were performed using a Rheometric Scientific STA 1500. Blank experiments were first run for all the as-received MgO samples using a heating ramp of 10 °C per minute from room temperature up to 800 °C in flowing nitrogen. After the blank experiments, all the as-received samples were activated to 300 °C prior to saturation at room temperature with CO_2_ for 30 minutes. After saturation, another heating ramp of 10 °C was used to heat up the samples up to 800 °C for the desorption experiments.

### 2.2 Ketonic decarboxylation reaction procedure

0.4004 g (2 mmol) of dodecanoic acid (TCI, 98%) were reacted at temperatures ranging from 250 to 300 °C with different catalyst loads of each of the different MgO samples. The amount of catalyst used varied between 5, 3 and 1% (w/w), with respect to the dodecanoic acid. The different samples were named using the letters UR as a prefix on the name to refer to the as-received un-reacted MgO powder. MgO 325 mesh (as-received, un-reacted MgO micron size: URMgO micro), MgO 100 nm (as-received, un-reacted MgO 100 nm size: URMgO 100 nm) and MgO 50 nm (as-received, un-reacted MgO 50 nm size: URMgO 50 nm). The carboxylic acid as well as the catalyst were put inside an autoclave (0.075 L Parr) using toluene (20 ml) as solvent (analytical grade, Fischer chemicals). Once sealed, the autoclave was purged with nitrogen four times to remove any trace of oxygen. Afterwards, the autoclave was heated to the desired temperature and the reaction run for one hour. Once the reaction time was over, the vessel was cooled and, once at room temperature, the crude product mixture was extracted.

### 2.3 Analysis of crude product

The extracted crude product was put into a 50 ml Falcon tube and centrifuged using a Beckman Coulter Avanti J-20XP centrifuge (1000 rpm, 30 min) to separate the solid catalyst. Catalyst material, spun to the bottom of the tube, was recovered to be further analysed using PXRD and FTIR spectroscopy. Afterwards, the crude product was analysed using a Shimadzu GC-2010 instrument with a flame ionisation detector (FID) with a HP-5 30 M length column, of 0.25 mm internal diameter and 0.25-micron film thickness coating. Before injection, a calibration curve using the internal standard method for the expected ketone was employed to perform the quantitative analysis (Fig. S1, ESI†).

### 2.4 Analysis of the MgO powders

For the spent catalyst (used for reaction at 300 °C), the letter S was used as a prefix before the name of the different spent MgO powders. MgO 325 mesh (spent MgO micron size: SMgO micro), MgO 100 nm (spent MgO 100 nm size: SMgO 100 nm) and MgO 50 nm (spent MgO 50 nm size: SMgO 50 nm). The spent catalyst from the 300 °C reactions, for all the different particle sizes, was recovered by centrifugation. Centrifuged samples were filtered to recover the wet catalyst, which was subsequently dried at room temperature for 1 hour and put inside an oven at 70 °C for 12 hours. Following this, the samples were put under high vacuum for 4 hours to remove any trace of the toluene solvent.

In order to determine the relative sorption of the dodecanoic acid on to the catalyst, the as-received MgO powders in all the different particle sizes (named URMgO), were contacted with dodecanoic acid dissolved in toluene using the exact same conditions as when the dodecanoic acid, catalyst and toluene were put inside the autoclave. Concentration of the dissolved dodecanoic acid was 0.1 M. The reaction proceeded at room temperature and at a stirring speed of 500 rpm for 12 hours. After completion, the samples were separated following the same methodology as the one mentioned above for the SMgO. These room temperature samples were named with the letters RT as a prefix before the name of each MgO sample. MgO 325 mesh (44 μm; MgO micron size: RTMgO micro), MgO 100 nm (MgO 100 nm size: RTMgO 100 nm) and MgO 50 nm (MgO 50 nm size: RTMgO 50 nm). The SMgO samples, as along with the RTMgO and URMgO samples, were analysed by TGA, PXRD and FTIR, as described above.

### 2.5 Preparation and analysis of magnesium dodecanoate

As a potential reaction byproduct analytical standard, and surface species on the MgO, magnesium dodecanoate (Mg dodecanoate) was prepared following a methodology described elsewhere.^16^ 10 g of dodecanoic acid (50 mmol) and 60 ml of water were heated and stirred inside a round-bottom flask in a bath at 80 °C until dodecanoic acid fully melted. Excess sodium hydroxide (NaOH, 100 mmol, 4 g) was added to the round bottom flask and leave it to react for 90 minutes. 10.1 g of MgCl_2_ (50 mmol) was added to the sodium dodecanoate slurry and left to stir over 60 minutes. Once the reaction was completed, sample was collected and washed with plenty of water and ethanol and left to dry at 70 °C for 12 hours. Magnesium dodecanoate was characterised through FTIR and PXRD.

## 3. Results

### 3.1 Magnesium oxide characterisation

**Fig. 1:**
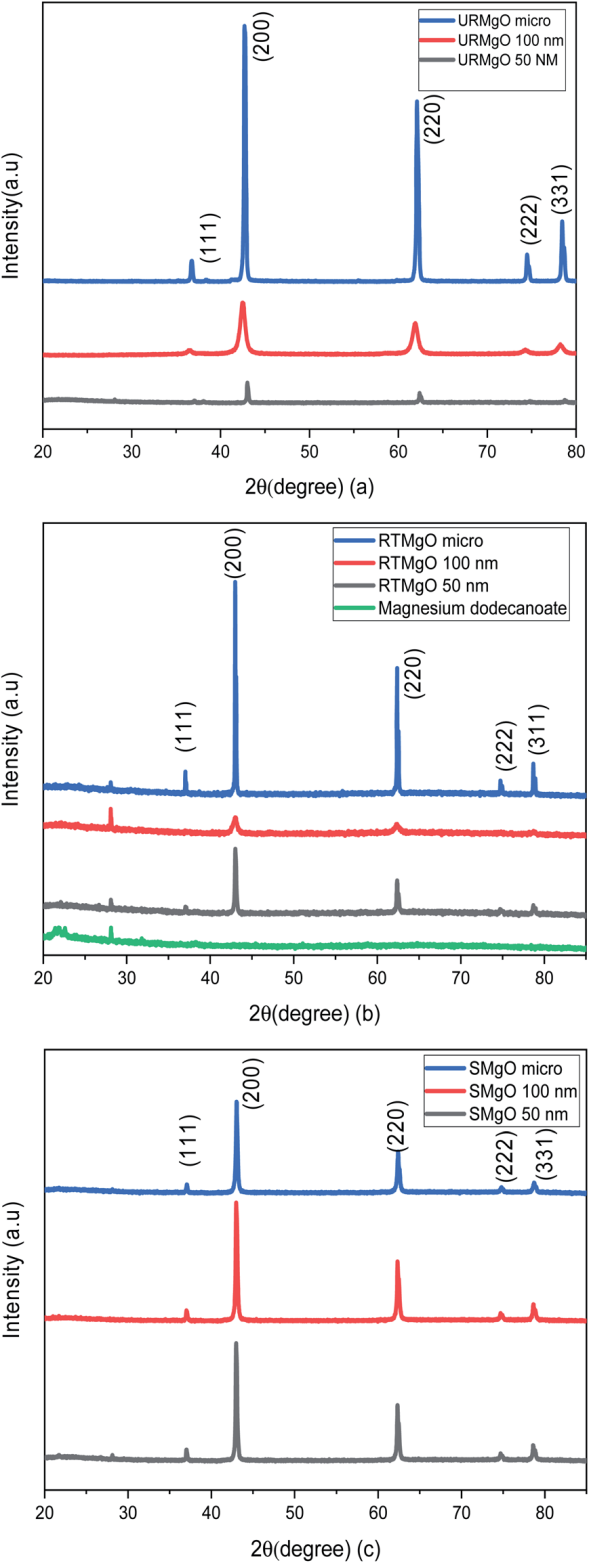
PXRD patterns of the different size MgO samples. (a) refers to the as received MgO samples (URMgO). (b) refers to the MgO samples exposed to the dissolved dodecanoic acid for 12 hours (RTMgO) and (c) shows the PXRD patters for the spent MgO, the samples of the oxide.

The PXRD patters for the URMgO, RTMgO and the SMgO samples are shown in Fig. 1(a), (b) and (c), respectively. Moreover, the peak width (FWHM) was calculated for all samples (Table 1) based on the strongest diffraction peak (200), *ca.* 43.03°.

**Table 1:** FWHM for all the MgO samples. Note that almost the same values of FWHM can be observed for the spent catalyst

FWHM	Sample
0.257	URMgO micro
0.744	URMgO 100 nm
0.224	URMgO 50 nm
0.168	RTMgO micro
0.688	RTMgO 100 nm
0.215	RTMgO 50 nm
0.268	SMgO micro
0.265	SMgO 100 nm
0.239	STMgO 50 nm

**Fig. 2:**
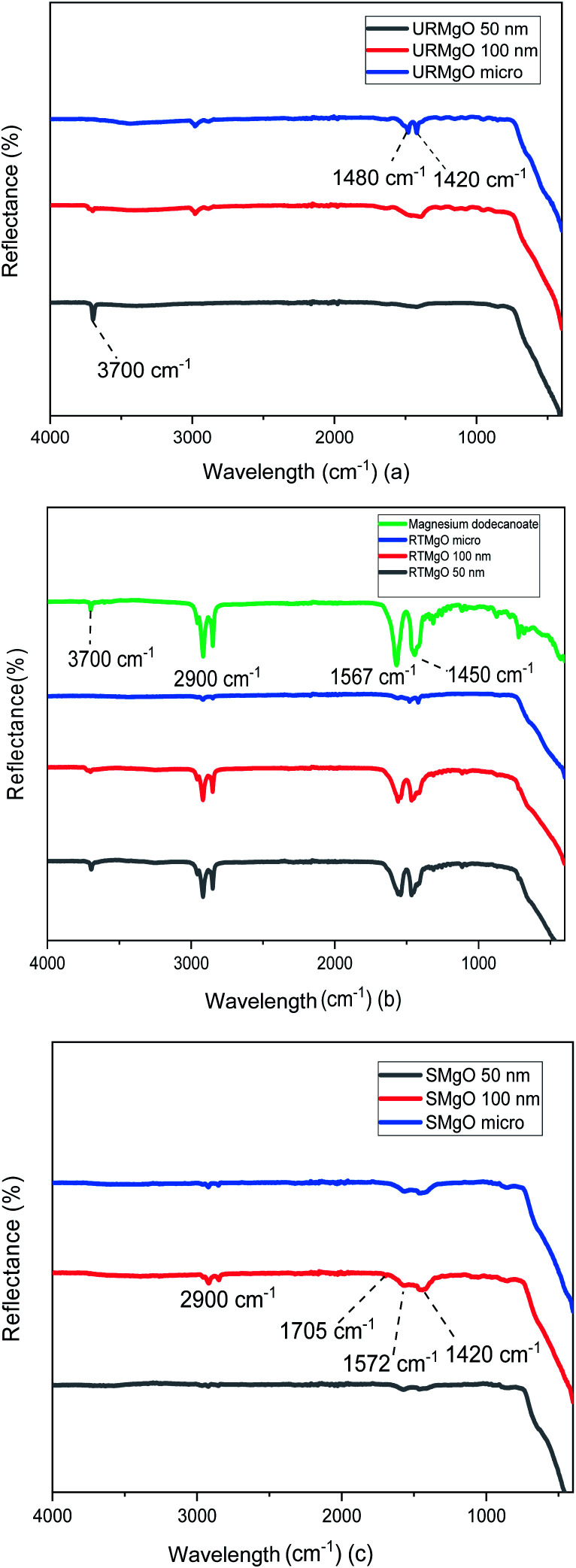
FTIR spectra of the URMgO, RTMgO and SMgO shown in (a), (b) and (c), respectively. (a) is the evaluation of the catalyst as-received, whereas (b) shows the catalyst after exposition with dodecanoic acid. (c) is the analysis of the post reaction MgO.

Fig. 1 shows typical sharp reflection peaks of cubic phase MgO, observed at *ca.* 43°, 62° and 74°, for all samples.^17,18^ However, for the MgO samples contacted with dodecanoic acid (RTMgO), an extra peak, at *ca.* 28° can be observed. The peak may arise owing to the formation of magnesium carboxylates.^18^ For comparison, the diffraction pattern for Mg dodecanoate is given in Fig. 1(b), which supports the latter, as the same peak at *ca.* 28 ° is observed for the Mg dodecanoate sample. Moreover, the formation of MgCO_3_ and Mg(OH)_2_ is expected for MgO samples when the latter are in contact with the environment. The presence of carbonate species for the URMgO and SMgO samples were further confirmed by the FTIR analysis, however, regarding the PXRD in Fig. 1(a) and (c), the presence of adsorbed CO_2_ species are barely visible, but can be assigned to the diffraction peaks *ca.* 28°.^19–21^ The PXRD patterns from the RTMgO samples and the SMgO ones are otherwise similar to the URMgO, this confirms no Mg(OH)_2_ was formed during the reaction. However, from Fig. 1(c), the post reaction SMgO samples present almost the same intensity principle XRD reflections across the three different particle sizes, also observed in Table 1, and the FWHM for the spent samples are almost equal to one another. This is different to the variation observed in FWHM for the URMgO and RTMgO samples, indicating recrystallisation of the MgO domains during the ketonisation reaction, as the crystallite domain size increase is dependent of the FWHM, according to the Scherrer equation.^22,23^ The FTIR spectra from URMgO, SMgO and RTMgO samples are shown in Fig. 2.

The characteristic vibration of the Mg–O bond is exhibited with a broad shoulder from around 470 cm^−1^ for all samples.^24^ For the URMgO samples (Fig. 2(a)), some amount of adsorbed atmospheric water is present in the form of rehydrated Mg(OH)_2_, with a clear peak at 3700 cm^−1^ for all three samples. Thus, the presence of peaks related to adsorbed atmospheric CO_2_ are observed around 1417–1420 cm^−1^ for the symmetric stretching of monodentate carbonate species, whereas other peaks can be encountered within the range of 1410 to 1480 cm^−1^. Those are assigned to the symmetric stretching of the bicarbonate species.^25^

In the case of the RTMgO samples, the spectra of the magnesium dodecanoate was added to the ones of the MgO, to be used as a point of comparison and to evaluate the formation of the carboxylate species over the surface of the material. As can be observed in Fig. 2(b), specifically for the Mg dodecanoate curve, the sharp peaks at 1567 and 1450 cm^−1^ could be assigned to the antisymmetric and symmetric stretching of the COO bonds.^26^ These stretching bands were also observed on all three RTMgO samples, indicating that the carboxylate species adsorbed over the surface of the catalyst. Moreover, the presence of stretching of the C–H bonds can be observed, which are usually encountered at around 2900 cm^−1^.^27^ A small 3700 cm^−1^ peak can be observed for all the samples in Fig. 2(b), indicating the presence of brucite (Mg(OH_2_)) in the material.

The samples corresponding to the SMgO (Fig. 2(c)) show small peaks at 1705 cm^−1^ and a broader one at 1572 cm^−1^, associated to the stretching of the CO bonds.^28–30^ Another set of small peaks from 1460 to 1420 cm^−1^ associated with stretching of C–H bonds, as well as the vibration of the C–O and C–C bonds, was present. No brucite was observed by FTIR in the SMgO samples, suggesting the temperature was sufficient to drive dehydroxylation.

**Fig. 3:**
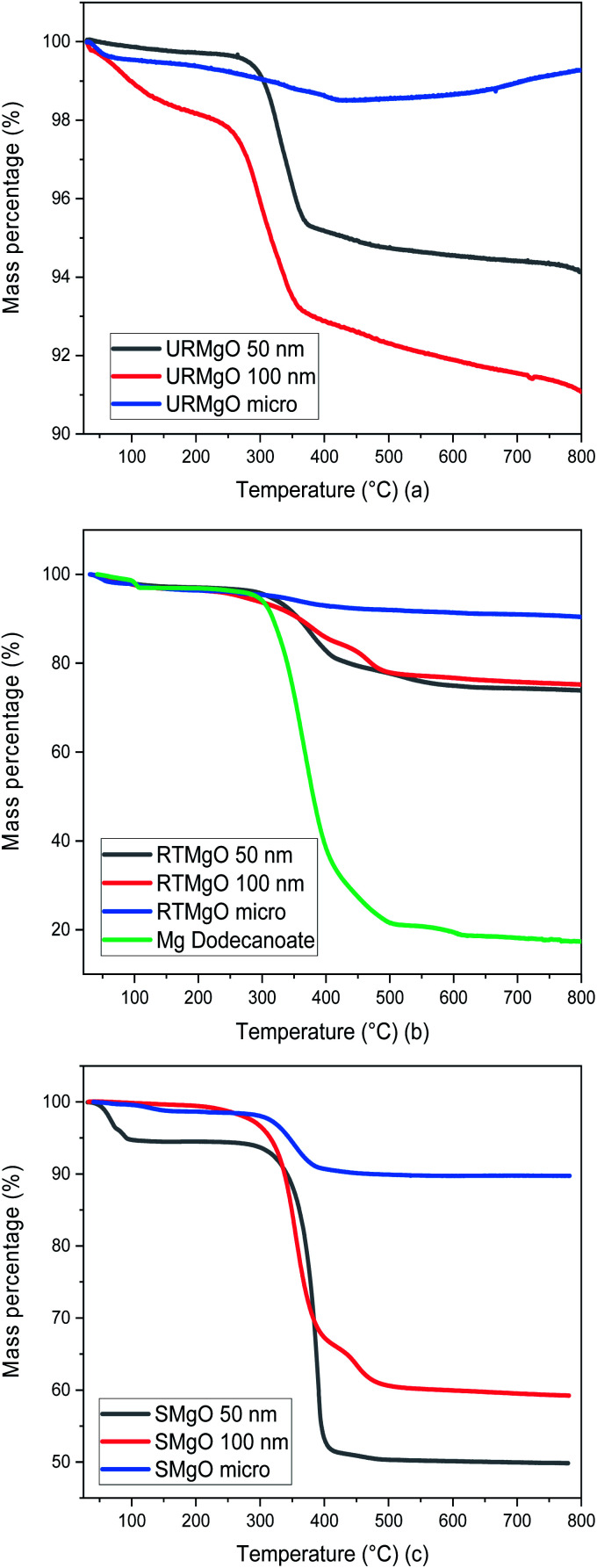
TGA graph for all the MgO samples. (a) is for URMgO, whereas (b) and (c) are for RTMgO and SMgO respectively.

Several TGA analysis were performed for all the catalyst samples. The results are shown in Fig. 3.

Although URMgO samples showed almost no adsorbed water, the presence of Mg(OH)_2_ is clear, as well as the presence of some adsorbed atmospheric CO_2_, with the latter confirmed by FTIR in Fig. 2(a) and by TGA, as observed in Fig. 3(a). Dehydroxylation (loss of the Mg(OH)_2_ species) is observed around 270 °C,^31,32^ followed by decarbonylation,^32,33^ causing a mass loss of up to 9%. Fig. 3(b) shows a two-step decomposition for the RTMgO samples. The first step could be attributed to dehydroxylation of the Mg(OH)_2_ species, followed by decomposition of the carboxylate species 290 °C up to 400 °C.^33,34^ The TGA curve of Mg dodecanoate showed similar decomposition onset to the rest of the RTMgO samples, supporting the suggestion that sorption of the acid occurs to the catalyst as an initial step. For RTMgO 100 nm, a small shoulder around 450 °C is observed, which could be attributed to some MgCO_3_ species formed due to the contact of the sample with environmental CO_2_. Subsequent degradation could be attributed to the complete decarbonylation of the catalyst, losing the carbonate to fully convert again into a metal oxide.^33,34^Fig. 3(c) shows a two-step decomposition for SMgO samples, with very little mass lost in the first step (water), and the second step attributed to the presence of adsorbed post-reaction ketone or residual reactant as observed in Fig. 2(c), removed from the catalyst once 400 °C is reached. No, or negligible, Mg(OH)_2_ was observed in the samples, as presented by Fig. 2(c), and no dehydroxylation occurs.

**Fig. 4:**
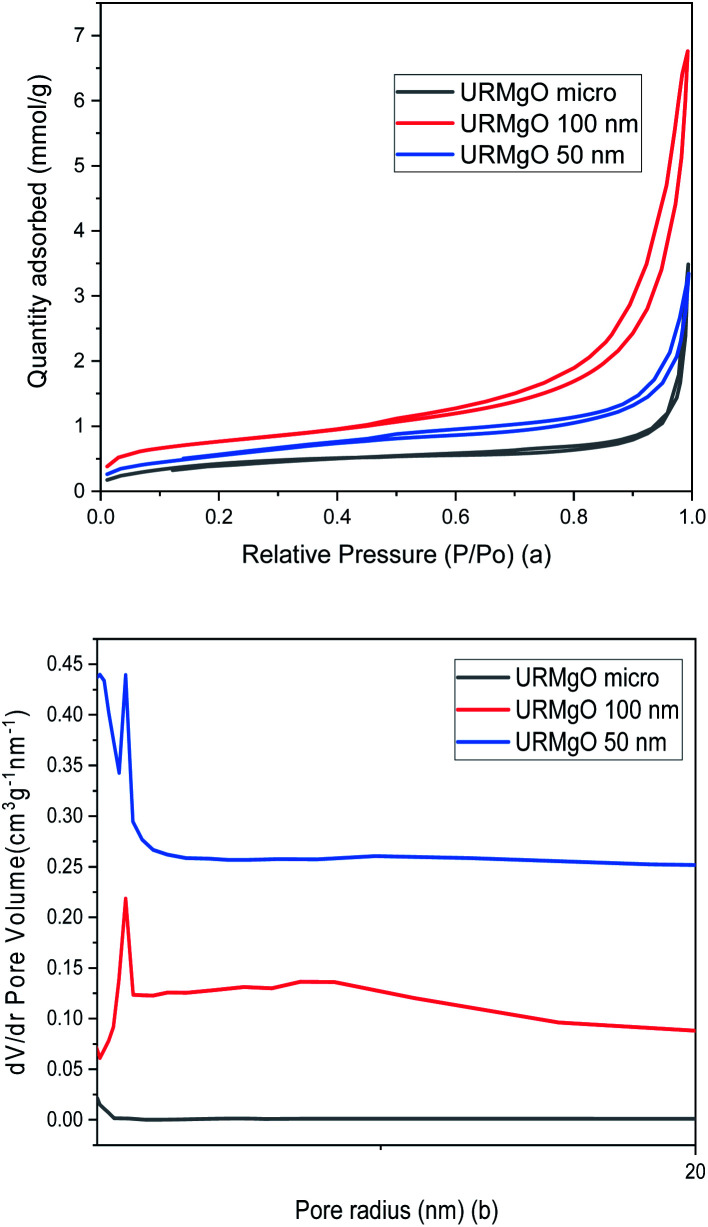
(a) Nitrogen adsorption/desorption isotherms and (b) pore size distribution of URMgO samples.

The surface area and pore analysis results for URMgO samples are given in Table 2. Nitrogen adsorption/desorption isotherms as well as the pore size distribution plots are shown in Fig. 4. For clarity, the Barret–Joyner–Halenda (BJH) plots for individual URMgO samples are provided in Fig. S2.† Quantification of the adsorbed amount of dodecanoic acid by the URMgO samples is presented in Table S1† as well as the calibration curve to calculate the aforementioned amount (Fig. S2†).

**Table 2:** Specific surface area results through BET and pore parameters results from BHJ method of the URMgO samples

Sample	Surface area (m^2^ g^−1^)	Pore volume (cm^3^ g^−1^)	Pore radius (nm)
URMgO micro	3.57	0.003	8.7
URMgO 100 nm	60.47	0.112	3.6
URMgO 50 nm	46.24	0.057	5.3

**Fig. 5:**
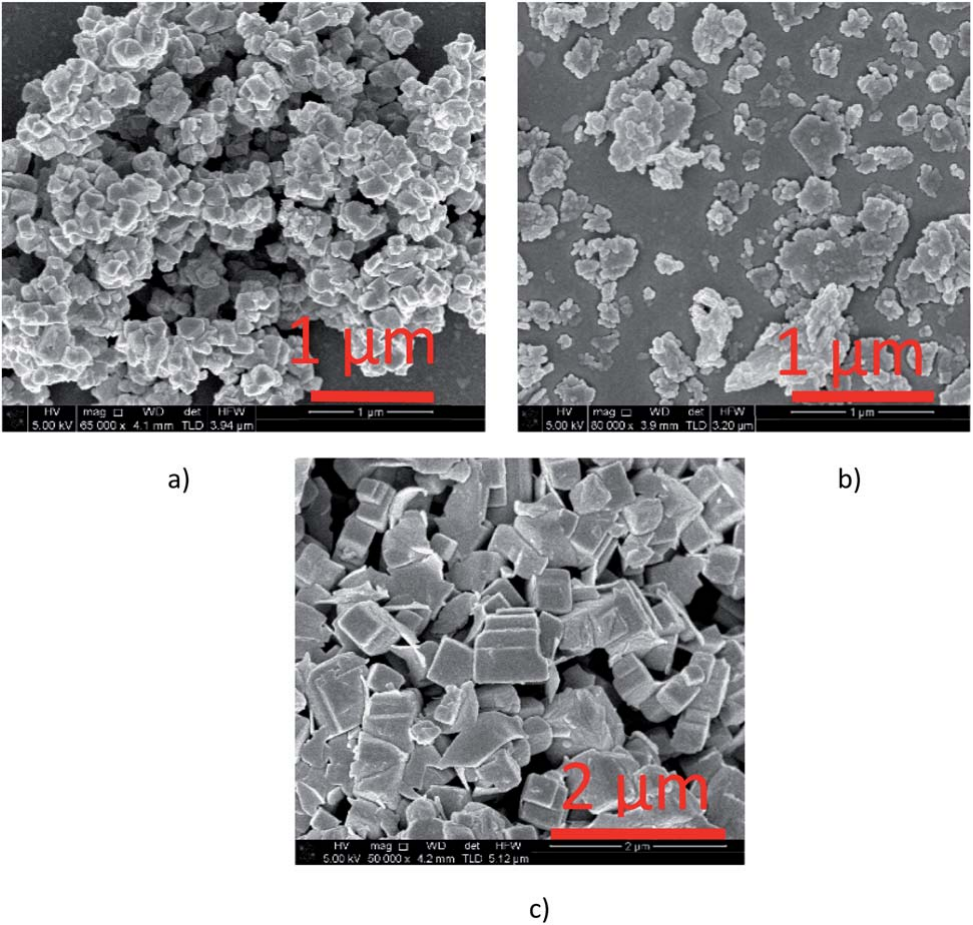
Scanning electron microscopy images of the different URMgO samples. More plate like aggregates can be observed for the nano sized materials ((a) URMgO 50 nm and (b) URMgO 100 nm) whereas the micro size MgO ((c) URMgO micro) presented the cubic particle morphology.

TPD analysis of the URMgO samples are shown in Fig. S3 of the ESI.† The behaviour of the material regarding the desorption of the CO_2_ molecules did not favour the quantification of the active sites, as neither the derivative of the TGA, nor the mass loss due to desorption could be calculated. SEM characterisation of the three URMgO samples are shown in Fig. 5. URMgO 50 nm, URMgO 100 nm and URMgO micro corresponds to Fig. 5(a), (b) and (c), respectively.

Agglomeration of the particles was observed for all the three samples, being more evident for the URMgO 50 nm and URMgO 100 nm, which also have clusters of spherical-like particles, whereas URMgO micro have a more cubic-like particle morphology.

### 3.2 Magnesium oxide as catalyst for the ketonic decarboxylation of dodecanoic acid

**Fig. 6:**
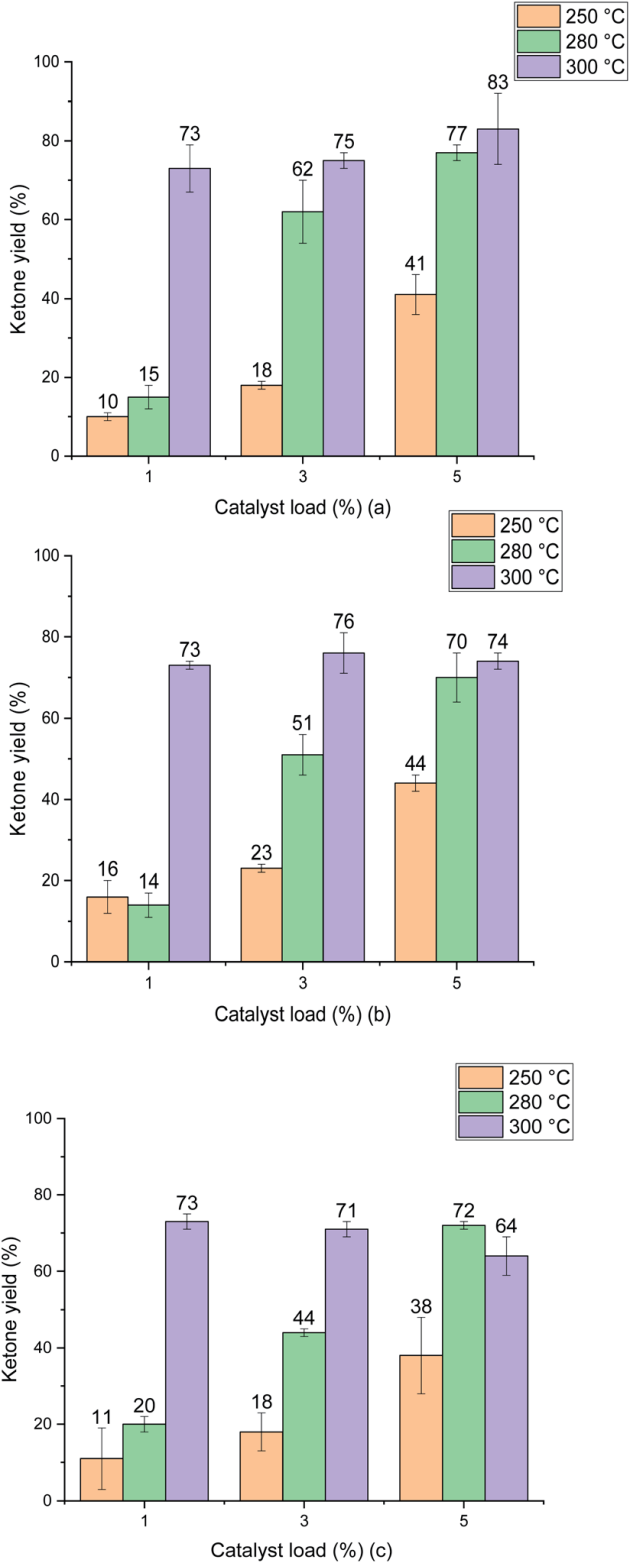
Ketone yield (12-tricosanone) product of ketonic decarboxylation of dodecanoic acid using the different catalyst samples. (a) shows the results from the URMgO 50 nm. (b) represents URMgO 100 nm and (c) represents URMgO micro. All samples shown a similar yield trend, and although the highest production was of 83% in (c), the differences from one another at different particles sizes are not high enough to assume the particle size could be making a significant impact to the yield.

The ketonic decarboxylation of dodecanoic acid was performed using different catalyst loads (1% w/w, 3% w/w and 5% w/w relative to reactant) of the as received MgO samples. The ketone yields produced by MgO 50 nm, MgO 100 nm and MgO micro are given in Fig. 6(a), (b) and (c), respectively. The reaction time was set to be one hour. Moreover, different temperatures were tested for this study, setting the reaction at 250 °C, 280 °C and to 300 °C. All experiments were performed in triplicate.

Fig. 6 shows that increasing the temperature resulted in increased yield for all the MgO particle sizes. It is also evident that the difference in particle size did not impact the yield of ketone significantly at the optimal temperature of 300 °C. Interestingly, the ketone yield remains the same for all catalyst loadings at 300 °C. The three different particle sizes yield similar results, irrespective of either the particle surface area (Table 2), particle size (Fig. 5) or the crystallite domain size of the starting material (URMgO), as given in Table 1.

It is important to notice that as no other product peak was observed during the post reaction analysis of the crude product mixture, selectivity towards the desired ketone product, 12-tricosanone, was assumed.

## 4. Discussion

In this section we analyse the production of 12-tricosanone from dodecanoic acid through ketonic decarboxylation using MgO of different particle sizes as the catalyst to promote the reaction. First, the characterisation of the different catalyst samples (URMgO, RTMgO and SMgO) is discussed prior to the evaluation of different reaction parameters (*e.g.* temperature, catalyst load and particle size).

### 4.1 Characterisation of the MgO catalysts at different process stages

#### 4.1.1 URMgO materials

XRD analysis of the URMgO show sharp and intense reflections corresponding to the MgO structure (Fig. 1(a)). URMgO-100 also shows some secondary reflections at *ca.* 38° and 59° two theta, which closely correspond with brucite (Mg(OH)_2_) reflections.^31^ The as received URMgO samples of different sizes, and particularly notable in the FTIR analysis (Fig. 2(a)) for URMgO 100 nm and URMgO micro, showed the presence of some adsorbed CO_2_, as carbonate, at *ca.* 1410 cm^−1^ to 1480 cm^−1^, related to the symmetric stretching of monodentate and bidentate species.^25^ Though no strong water adsorption peak was observed at *ca.* 3500 cm^−1^, the brucite characteristic adsorption at *ca.* 3700 cm^−1^ is also observed in Fig. 2(a).^31^ This is clear indication of some rehydration of MgO to Mg(OH)_2_. The latter was further investigated using the TGA analysis, given in Fig. 3, which showed little surface bound water, with <1% for URMgO micro and URMgO 50 nm, and slightly more (∼2% mass) water on URMgO 100 nm. TGA analysis also evidenced the existence of some brucite phase in the starting material for the smaller particle sizes, with both URMgO 50 nm and URMgO 100 nm showing characteristic losses through dehydroxylation at an onset temperature of *ca.* 280 °C. At above this temperature, all materials showed a small 1–2% mass loss, presumably through loss of the carbonate fraction evidenced in the FTIR (Fig. 2(a)). It should be noted that the higher adsorption of carbonate by URMgO 100 nm and URMgO micro could be attributed to the samples being older batches than URMgO 50 nm, therefore, they have been more in contact with atmospheric carbon dioxide and water than the latter, forming Mg(OH)_2_ and MgCO_3_, which is in agreement with the TGA plot in Fig. 3(a). The URMgO samples were left to dry at 200 °C inside an oven for 24 hours and TGA was performed again, however, the behaviour of the mass loss curves were the same as the ones presented in Fig. 3(a). The SEM analysis in Fig. 5 showed differences in morphology between the nanoparticles (*i.e.*, URMgO 50 nm and URMgO 100 nm) and URMgO micro. Nanoparticles showed spherical agglomerates of plate-like particles whereas URMgO micro crystals have cubic morphology. Specific surface area, given in Table 2, and crystallite domain size (from FWHM, Table 1), do not follow a trend according to particle size.

#### 4.1.2 RTMgO materials

The XRD patterns for the RTMgO (Fig. 1(b)) did not change significantly, when compared to the URMgO, though the presence of a minor phase, possibly owing to magnesium-dodecanoate complexes, was observed. The latter could be confirmed as magnesium dodecanoate was prepared as a reference compound and used for comparison, as observed in Fig. 1(b). Nevertheless, and contrary to what is pointed out by Mekhemer *et al.*^30^ the bulk structure of the MgO was unchanged when in contact with a carboxylic acid at room temperature. FTIR results, presented in Fig. 2(b), showed that after contact with dodecanoic acid the RTMgO samples all showed characteristic C–H stretching modes at *ca.* 2900 cm^−1^, showing the MgO adsorbs dodecanoic acid. C–H peaks associated with toluene could be discounted as the samples were dried and put under vacuum, as described in Section 2.4. This was further confirmed by the characteristic symmetric/anti-symmetric stretching of the carboxylate group at 1450 cm^−1^ and 1567 cm^−1^, respectively.^26^ The latter were also compared with the spectra obtained from the reference compound Mg-dodecanoate, which showed the same trend, as observed in Fig. 2(b), which confirmed the presence of the carboxylate species. The TGA thermograms in Fig. 3(b) showed a strong surface area effect for the adsorption of the organic molecules, with the low surface area (3.57 m^2^ g^−1^) RTMgO micron showing a ∼5% mass loss above 300 °C, whereas the high surface area RTMgO 100 nm (60.47 m^2^ g^−1^) and RTMgO 50 nm (46.24 m^2^ g^−1^) show mass losses of ∼19%, and 22% respectively, though including any small dehydroxylation contribution as noted above. The presence of a small shoulder at 450 °C for RTMgO 100 nm could be attributed to the formation of MgCO_3_ as a result from the adsorption of environmental CO_2_. The FTIR and TGA also indicate the presence of adsorbed water in the form of Mg(OH)_2_ at *ca.* 3700 cm^−1^. The FWHM (Table 1) for the RTMgO samples also closely follows those of the URMgO, indicating similar crystalline domain sizes within the particles.

#### 4.1.3 SMgO materials

Following the reaction process, the recovered MgO materials (SMgO) were analysed again to allow comparison with the RTMgO. SMgO were evaluated from the other two temperatures (250 °C and 280 °C) to find if their bulk structure suffered any change as, for example, Snell & Shanks found for ceria at a specific temperature at the onset of ketonic decarboxylation.^13^ However, this was not the case. None of the samples showed significant changes to the XRD patterns reported in Fig. 1 (see ESI†), though the different particle size patterns now seem more uniform, suggesting a degree of recrystallization has occurred at the higher temperatures used. The latter argument is in agreement with the FWHM data obtained in Table 1 for all the MgO samples, with the SMgO showing very similar crystal domain sizes across the different particle sizes relative to the URMgO and RTMgO samples, where crystal domain sizes vary significantly according to particle size.

This supports the hypothesis than the ketonic decarboxylation reaction happens to occur purely as a surface mechanism rather than affecting the bulk structure of the MgO, in contrast to the work of Pestman, *et al.*^35^ in which ketonic decarboxylation was postulated to only occur over the surface of the catalyst at nonstationary reaction conditions and a possibly bulk carboxylate restructuring of the latter occurring at stationary conditions. TGA analyses were also used to validate the information obtained through the PXRD (Fig. 1(c)) and FTIR analysis (Fig. 2(c)), in which decomposition curves from the different MgO samples were observed (Fig. 3(c)).

SMgO samples show the presence of an FTIR adsorption at *ca.* 1740 cm^−1^, indicative of ketone C=O stretching,^30^ not present in the RTMgO samples. The relative intensity of the carboxylate symmetric and anti-symmetric bands, as compared to C–H stretching intensity, are also reduced in the SMgO samples, particularly in the high surface area nanoMgO, confirming the conversion of the dodecanoic acid starting material to the product. The FTIR of the SMgO samples shows no broad water adsorption band at *ca.* 3500 cm^−1^, as might be expected post heating in a non-polar solvent, though the TGA trace shows the SMgO 50 nm material to have picked up water during sample preparation. The % of material lost during pyrolysis in the TGA from 300 °C to 500 °C, indicating sorbed reactant/product, was close to 8%, 40% and 42% for the SMgO micro, SMgO 100 nm and SMgO 50 nm samples, respectively. This is approximately double than observed for the comparative samples treated at room temperature and, as for the RTMgO samples, a strong surface area effect is notice with the nanometer sized samples holding approximately five times the organic matter (wt%) compared to the MgO micro.

In summary, the two roughly comparable URMgO 50 nm and URMgO 100 nm samples appear to show a strong surface area determined adsorption effect both pre- and post-reaction. If the active sites are of similar strength and the reaction is controlled by number of active sites available, we would expect to see a significant difference in yield for these two samples when compared to the URMgO micro. Through TPD-CO_2_ desorption, a quantification of the active sites on the URMgO samples could be achieved, to further measure the basicity strength. Moreover, the plots, as observed in Fig. S3,† provides some insights regarding a possible crystallization of the material *circa* 310 °C (the exothermic peak observed in the DSC plots for all the URMgO samples) which seems to agree with the results reported in Table 1 regarding the FWHM of the SMgO. Thus, the behaviour of the URMgO adsorbing CO_2_ through the TPD analysis, as observed in Fig. S3,† seems similar, with all the basic sites within the samples being of similar strength and quantity. In the next sections we explore the impact of various parameters on reaction yield for a fixed reaction time of 1 h.

### 4.2 Effect of temperature on ketonic decarboxylation over MgO

Test reactions were undertaken with dodecanoic acid dissolved in toluene, at the three different reaction conditions of 250 °C, 280 °C and 300 °C, and with no MgO catalyst present, to ascertain whether any thermal conversion occurred. No appreciable formation of the desired ketone product, 12-tricosanone, was observed in these tests. As observed in Fig. 6, when the MgO catalyst was added to the dissolved dodecanoic acid, the desired ketone produce was obtained.

From Fig. 6 it is apparent that for the two lower temperatures used, the yield of 12-tricosanone was dependent on catalyst load. When temperature was increased from 250 °C to 280 °C, an increase on the yield of the ketone was observed. This suggested that either (i) the reaction was controlled by the availability of certain active sites in the catalyst, which are temperature dependent, or (ii) the temperature was approaching the activation energy for the α-hydrogen abstraction from the carboxylate acid necessary for the ketonisation reaction,^9,10,13^ and the turnover frequency was limited at the catalyst base sites. It is notable that at *circa* 280 °C in the TGA analysis in Fig. 3, the onset of dehydroxylation of any hydrated MgO is observed.

When a reaction temperature of 300 °C was used, the highest yield of ketone (>70%) was observed in all but one case (mean of 64%) and, even allowing for the different catalyst loads and particle sizes used, the 12-tricosanone production was not significantly different from one another. If the reaction temperature was increased above 300 °C, a whole set of different aromatic products started to appear within the post-reaction analysis, probably by a pyrolytic degradation effect of the products and, also *via* potential reactions with the solvent.^30^ PXRD analysis (Fig. 1) of the post reaction catalyst (SMgO) shows the catalyst was largely unaltered. As Mekhemer, *et al.*^30^ noted, and suggested here by the post-reaction analysis of the spent catalyst (SMgO) by FTIR (shown in Fig. 2(a)), some ketones remained adsorbed to the MgO surface, which due to coordination with Lewis acid sites,^28,29^ could be activated at high temperatures to undergo further reactions. The TGA thermograms in Fig. 3 shows that, for the URMgO, at 300 °C, MgO fully dehydroxylated, and the recycling of active basic Mg_3_OH to Mg_3_O^−^ sites was rapid.

### 4.3 Effect of the catalyst loading for the ketonic decarboxylation of dodecanoic acid

As explored in Section 4.1, it can be inferred from Fig. 6 that there is a relationship between reaction temperature and catalyst loading, a directly proportional relationship of yield, temperature and catalyst load up until 300 °C. For all the MgO samples used during the ketonic decarboxylation experiments, when working at temperatures below 300 °C, it was required to increase the catalyst load with respect to the feed of dodecanoic acid to increase the production of the 12-tricosanone. At 250 °C, the temperature at which ketone production was started to be observed, 1% (w/w) of the catalyst seemed insufficient to generate a ketone yield above 10%, however, when increasing the loading up to 5% (w/w), a ketone yield of around 45% was obtained. The same was true when working at 280 °C, increasing the catalyst load directly impacted the ketone production. However, once the temperature reached 300 °C, the catalyst loading did not affect the ketone yield, as with as low as 1% (w/w) for all the MgO samples, the ketone yield was similar from one another (around 75%). As discussed in Section 4.2, once the right temperature for the carboxylates to be activated was achieved, it could be assumed that these will react and so form 12-tricosanone, forming CO_2_ and H_2_O.^33^ Raising the catalyst load increases the available surface area and number of catalytic sites, which may be a critical parameter for increased reactivity. If so, moving from large particle size to a small particle size for a given catalyst loading should give similar effects and this is considered in the next section.

### 4.4 Effect of the particle size of different MgO powders for the ketonic decarboxylation of dodecanoic acid

When the catalytic activity of all the MgO powder samples were tested one against other, the three different particle sizes presented similar results, with no significant yield difference between one another, according to Fig. 6. Nevertheless, it is worth noticing that there is indeed a minor positive particle size effect when evaluating the ketonic decarboxylation reaction at the highest temperature and catalyst load (300 °C and 5% (w/w), respectively). During the realisation of the experiments, it was expected that the smallest particle size MgO powder (URMgO 50 nm) would produce a significant amount of more ketone than the rest of the samples (URMgO 100 nm and URMgO micro) as the smaller the particle size the more surface area and therefore better results are expected for any heterogeneous catalyst. However, that was not the case. The yields stayed relatively similar between all the samples (∼75–80%, considering the standard deviation) when evaluated for all the ranges of catalyst loads (1 to 5%) and temperatures (250 to 300 °C). It can be noted, due to the latter results that, if the reaction was controlled by the number of active sites over the catalyst's surface area (see Table 2 for specific surface area measurements), a notable difference between the yields of 12-tricosanone would be observed when using the nano MgO samples compared when using the micro size one. This indicates that, rather than controlling the reaction, the surface area effect is not directly related to the amount that could be produced when using an oxide for ketonic decarboxylation of fatty acids. As observed in Fig. 1(a), decreasing the crystalline domain size results in broadening of the peaks. Nevertheless, when the FWHM of the reacted MgO catalysts (SMgO) are compared (in Table 1) it can be seen that all the different particle size MgO give a similar crystallite domain size post reaction, suggesting recrystallisation is happening at 300 °C. This now explains the similar reactivity, with the crystal domain size controlling conversion and this becomes similar across all the MgO, irrespective of the initial particle size, under reaction conditions.^22,23^ Interestingly, Fig. 2 shows a strong inverse correlation between particle size and the amount of mass loss in the RTMgO samples, relating to fatty acid sorption, and in the SMgO samples, indicating surface area plays a role in the reaction process during sorption of the reactants.

### 4.5 MgO as an effective catalyst to promote ketonic decarboxylation

The studies from Corma, *et al.*^14^ and Mekhemer *et al.*^30^ provided data regarding the effectivity of MgO powder to promote ketonic decarboxylation of carboxylic acids. Our recent studies^15,32^ with a different type of material, the layered double hydroxides, also provided good ketone yields, with the catalytic activity being probably promoted by mixed basicity sites on the brucite-like layers.^15,36,37^ As mentioned in Section 4.4 and as observed in Fig. 1(c) and Table 1, there is a clear relationship between the crystallinity of the MgO samples and the reactivity of the latter. The MgO samples reaching a similar level of crystallinity post-reaction (Table 1) and producing similar ketone yields at 300 °C (Fig. 6) indicates that the temperature modified the crystal structure, making the SMgO of similar narrowness and crystallite domain sizes (Fig. 1(c)), which suggest the latter are responsible for the catalytic activity of the MgO during the ketonisation reaction, rather than the textural properties of the catalyst.^38^ Moreover, and as mentioned in Section 4.2, the active sites cannot be discarded and should be credited as actors promoting catalytic activity within the MgO samples used in this study. As the literature suggests,^39,40^ in condensation-like reactions, the presence of water and the subsequent hydroxylation of the surface is key in enhancing the reactivity of the MgO basic sites. Considering the latter, it seems likely that besides low coordination O^2−^ promoting the deprotonation of the adsorbed carboxylic acids to convert them into carboxylate species that will further undergo ketonic decarboxylation through a β-ketoacid as recent studies suggest,^10,41^ OH species adsorb over the surface of the catalyst once water is generated. This would hydroxylate the surface of the MgO, and although poisoning of some of the low coordination O^2−^ by hydrogens might occur, the number of active sites might remain constant due to the presence of the OH species, which are of higher reactivity than the former.^42^ The latter OH reactivity has been observed in hydrotalcite materials^43^ and in hydrotalcite-like materials (*i.e.*, layered-double hydroxides) such as in our last study.^15^ The Brønsted basicity of the OH species will further deprotonate a carboxylic acid molecule leading to the formation of a likely reactive intermediate which then will react, followed by desorption of the desired ketone products. The latter assumption arise from the study of Bailly *et al.*^42^ in which it was probed that Brønsted basicity of MgO surfaces decreased when they were hydroxylated, but the latter was compensated by the highly reactive hydroxyl groups over the surface of the oxide.

## 5. Conclusion

Ketonic decarboxylation of fatty acids has become an extensively explored research topic. Here, the possible impacts of the particle size within the reaction conditions as well as small amounts of catalyst were tested for the ketonic decarboxylation of dodecanoic acid, with high ketone production at moderate temperatures using the inexpensive catalyst MgO.

It was observed that the reaction temperature is an important reaction parameter, as it was observed that for temperatures below 300 °C, more catalyst is needed to increase the yield of ketone. However, once 300 °C was reached, up to 1% (w/w) of the catalyst delivered ketone yields above 70%. Although it could be expected that the presence of more active sites over the surface of the smallest particle size catalyst would impact the ketone yield positively, both, the nano and the micro size MgO delivered similar results at 300 °C, even at different catalyst loads. The analysis performed using the spent catalyst (SMgO) suggest that recrystallisation occurs at 300 °C, with the crystallite size being a key parameter for the ketonic decarboxylation reaction. Overall, increased surface area and active site availability, whether through varying particle size or catalyst loading, were found to have less impact on reaction yield than the temperature.

## Conflicts of interest

There are no conflicts to declare.

## Supplementary Material

RA-011-D1RA06871G-s001
