# Supplementary material for: Heterogeneous ketonic decarboxylation of dodecanoic acid: studying reaction parameters
Source: RSC Adv. 2021 Nov 3;11(56):35575–84. doi: 10.1039/d1ra06871g (PMC9043230; doi:10.1039/d1ra06871g)
Supplement: RA-011-D1RA06871G-s001 [file RA-011-D1RA06871G-s001.pdf]

Electronic Supplementary Material (ESI) for RSC Advances.
This journal is © The Royal Society of Chemistry 2021

Supporting information

Heterogeneous ketonic decarboxylation of dodecanoic acid: Studying reaction parameters

Diego D. Perera-Solis[a,b], Vladimir L. Zholobenko[c], Andrew Whiting[a,b] and Hugh Christopher Greenwell[a,d*]

[a.] *Department of Chemistry, Durham University, Durham DH1 3LE, UK. diego.d.perera-solis@durham.ac.uk*
[b.] *Centre for Sustainable Chemical Processes, Department of Chemistry, Durham University, Durham DH13LE, UK*
[c.] *Lennard-Jones Laboratories, Keele University, Staffordshire ST5 5BG, UK; v.l.zholobenko@keele.ac.uk*
[d.] *Department of Earth Sciences, Durham University, Durham DH1 3LE, UK*

Table S1. Amount of adsorbed dodecanoic acid in solution. The solution was left stirring for 1 hour for a subsequent centrifugation to separate the wet catalyst, followed by a drying at an oven over night at 70 ℃.

Dodecanoic acid added (g)	Grams adsorbed (g)	Entry (sample)
0.4019	0.031378654	RTMgO 50 nm
0.419	0.028536467	RTMgO 100 nm
0.4073	0.023337022	RTMgO micro

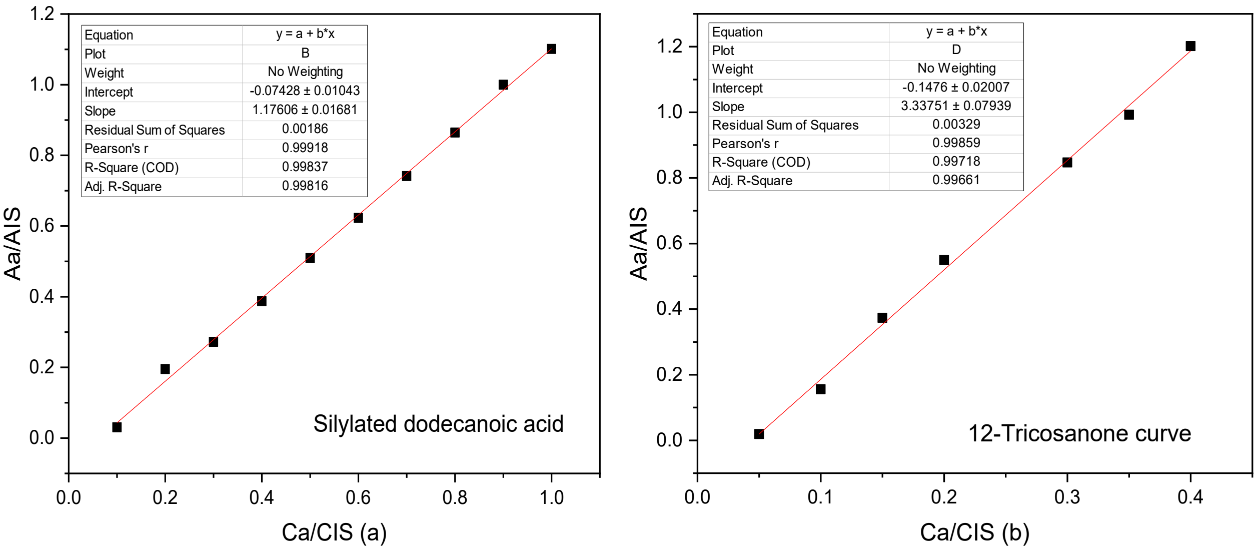

Fig. S1 a) Calibration curve of the silylated dodecanoic acid and b) calibration curve of the 12-tricosanone

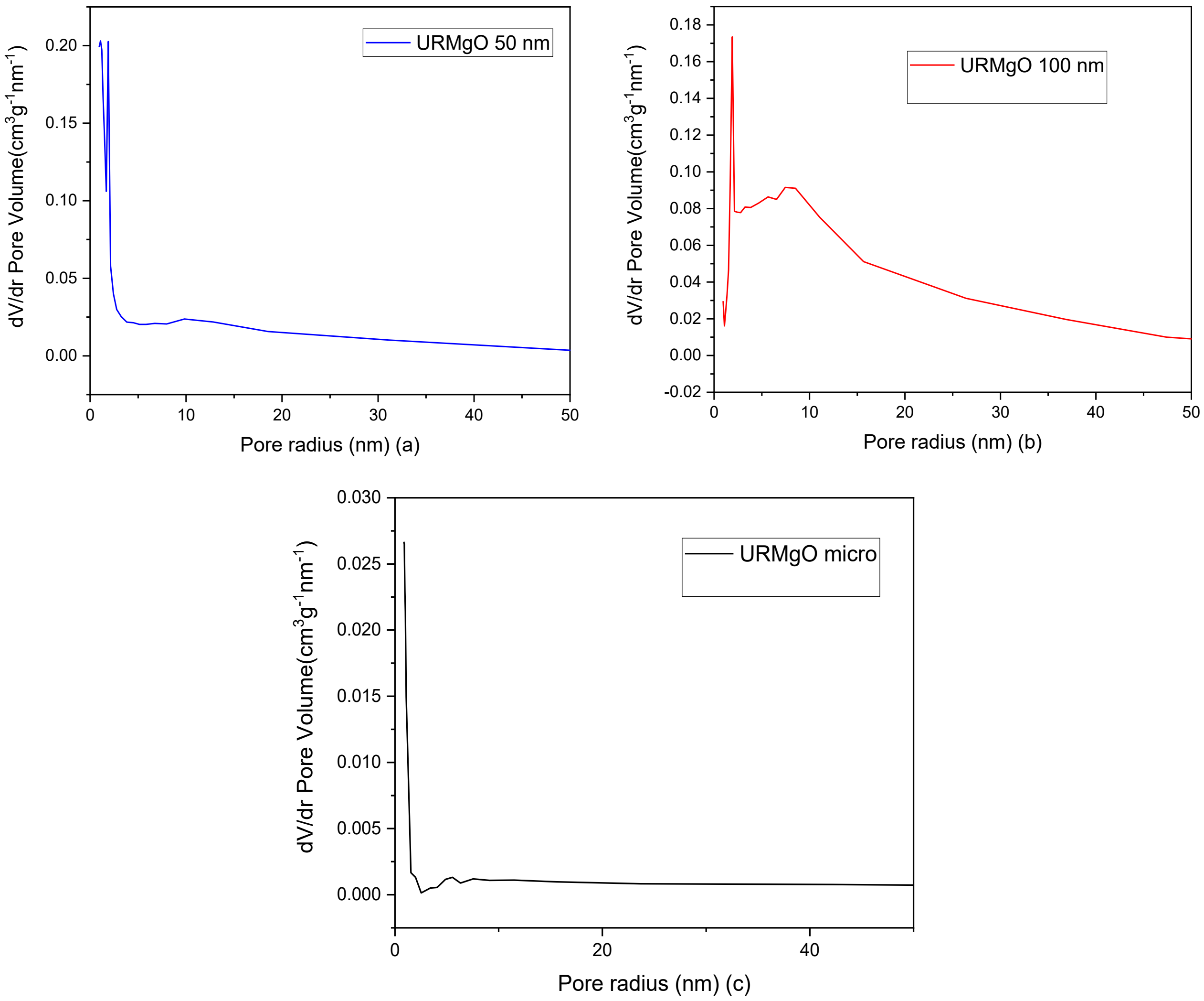

Fig. S2 Scaled Barret-Joyner-Halenda plots from the URMgO samples. a) Refers to the URMgO 50 nm. b) Refers to the URMgO 100 nm and c) refers to the URMgO micro. The plots were put together in a separate figure as in Fig. 4 the micro and mesoporous can not be distinguished.

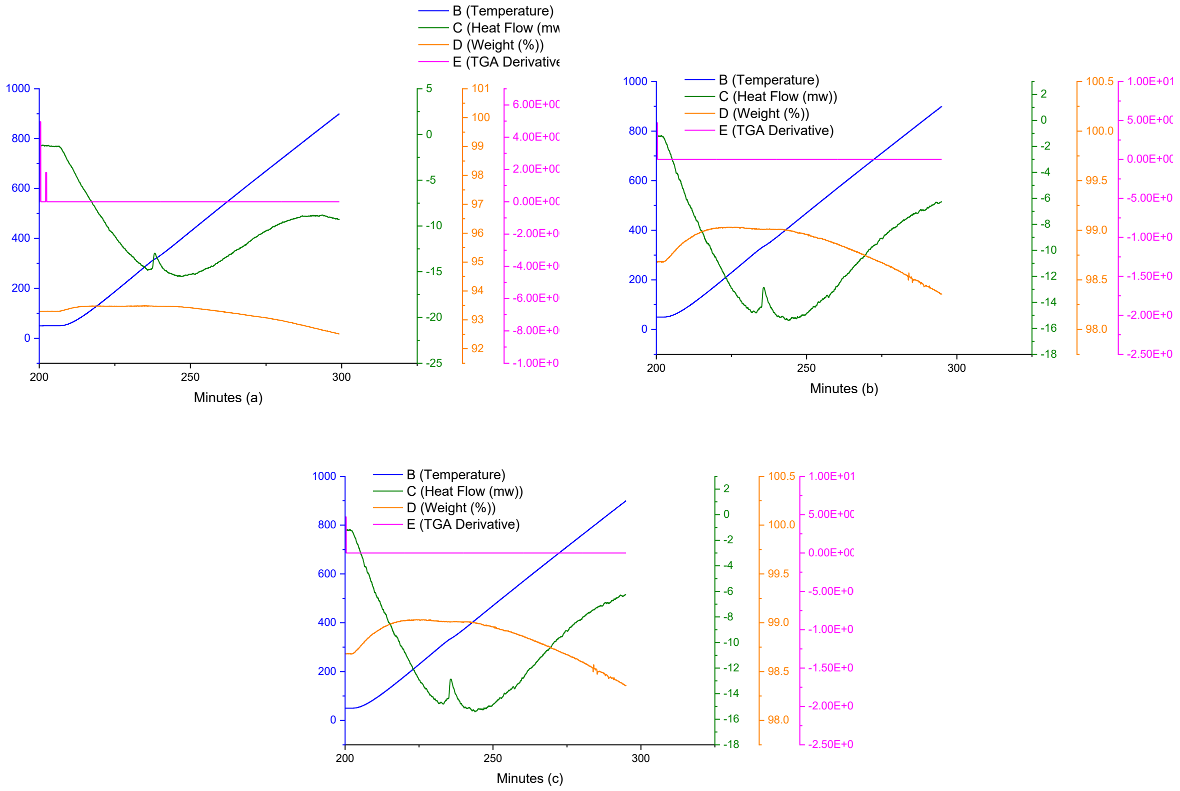

Fig. S3 Temperature programmed desorption curves from the URMgO samples. Figure S2(a) represents URMgO 50 nm, whereas URMgO 100 nm and URMgO micro are Figure S2(b) and Figure S2(c), respectively.
